# Implementation of perinatal mental health screening for parents of infants in a level IV neonatal intensive care unit: A quality improvement initiative

**DOI:** 10.1038/s41372-025-02315-z

**Published:** 2025-05-07

**Authors:** Sarah A. Swenson, Megan E. Paulsen, Kelsey Carrigan, Rachael Stover-Haney, Delaney Wilton, Brittney Skalland, Andrea L. Lampland, Ellen Diego, Maria Kroupina, Erin A. Osterholm, Ann Downey

**Affiliations:** 1 University of Minnesota Medical School, Department of Pediatrics, Division of Neonatology, Minneapolis, MN USA; 2 University of Nebraska Medical Center, Department of Pediatrics, Division of Neonatology, Omaha, NE USA; 3 Children’s Minnesota, Department of Neonatology, Minneapolis, MN USA; 4 MHealth Fairview Masonic Children’s Hospital, Minneapolis, MN USA; 5 University of Minnesota Medical School, Department of Pediatrics, Division of Clinical Behavioral Neuroscience, Minneapolis, MN USA

**Keywords:** Paediatrics, Health services, Depression

## Abstract

**Objective:**

We aimed to establish standardized perinatal mental health (PMH) screening performed by social workers for parents in the neonatal intensive care unit (NICU) at 1, 2, 4, and 6 months, increasing screening rates from 0% to 70% within 6 months.

**Study design:**

Baseline data evaluated informal PMH assessments. Primary measure was percent of parents screened and was monitored by statistical process control charts. Process measures were percent of parents with scores above threshold for referral for further evaluation and/or treatment, appropriately referred, and declining screening. Balancing measures were negative perceptions of screening.

**Results:**

The centerline for screening rate was 80% for mothers and 72% for partners. Screening increased concerns detected beyond 1 month from 12 to 60. Concerns representing partners increased from 3/52 (6%) to 18/60 (30%).

**Conclusion:**

Standardized NICU PMH screening improved identification of PMH concerns beyond the first weeks of admission for both mothers and partners.

## Introduction

Perinatal mental health (PMH) disorders are the most common complication of childbirth and the leading cause of preventable maternal postpartum mortality [1]. Identification and treatment of PMH disorders, such as perinatal anxiety and depression, improve the long-term health of children [2]. Infants hospitalized in the neonatal intensive care unit (NICU) are at higher risk for long-term health complications, and parents of infants impacted by the NICU have higher risk of PMH disorders, many of which are underdiagnosed and therefore remain untreated [3, 4]. Timely identification and treatment of PMH concerns in NICU parents has the potential to yield substantial benefits for both infants impacted by the NICU and their families.

Recommendations for PMH screening for NICU parents have evolved in both the inpatient and outpatient settings. In 2015, the NICU Mental Health Professional Workgroup, which included parents of infants impacted by the NICU, recommended evaluating families for all forms of emotional distress using standardized, validated tools [5]. Recognizing the prevalence and impact of PMH on family wellbeing and child outcomes, the American Academy of Pediatrics (AAP) issued a policy statement in 2019 calling for longitudinal maternal postpartum depression screening at the 1-, 2-, 4-, and 6-month well child visits with a consideration for screening partners at 6 months [2]. However, many infants remain hospitalized at these intervals, and their parents therefore miss opportunities for PMH screening and early referral, despite being at higher risk [6–8]. Most recently, the AAP included depression screening for all families in their standards for neonatal care at level II, III, and IV NICUs [9]. Parents impacted by the NICU have separately called for routine PMH screening [10] as well as research to improve support for and understanding of parent PMH needs [11], and parents’ ongoing partnership in creating effective PMH screening programs is critical.

As the AAP standards of neonatal care highlight [9], all parents can experience PMH concerns regardless of gender identity or gestational status (GS). Experts in paternal PMH as well as mothers have highlighted the lack of routine screening for fathers [12, 13], and the National Perinatal Association recommends screening fathers for depression at least twice before a child’s first birthday [14]. This is especially important in the NICU, where studies have shown that although mothers and fathers are affected by PMH concerns at similar rates, fathers are less likely to access support [15]. While most research on PMH discusses parents in terms of mothers and fathers, we recognize that more inclusive language is necessary to include LGBTQ+ parents and have therefore chosen to use mothers and partners, but we recognize that this language may not describe all families.

Initiatives to implement PMH screening within the NICU [16–21], reviews of these projects [22, 23], and best practice guidelines by groups with embedded psychology support [24] have been published. However, an effective, standardized way to screen both mothers and partners for PMH disorders longitudinally has not been determined for the NICU population across systems with varied degrees of psychosocial support. Thus, while the AAP recommends depression screening as standard of care [9], guidance is needed to inform screening tools, cadence, referral thresholds, and inclusivity of screening, and further input from parents is critical to ensure that screening meets their needs. The objective of this study is to use quality improvement (QI) science to inform these gaps and highlight areas for future research.

## Methods

### Rationale

Our global aim was to improve PMH support for families impacted by the NICU through a combination of PMH screening, educational interventions, integration of PMH support across disciplines, and feedback from families. As an initial step, we used QI science to implement standardized, longitudinal PMH screening performed by maternal child health social workers (MCHSW) using the Edinburgh Postnatal Depression Scale (EPDS) and anxiety subscale (EPDS-3A) [25]. Through standardized, longitudinal PMH screening, we endeavored to improve effectiveness in detecting PMH concerns, promote timely referrals, and improve equity in detecting PMH concerns in partners.

Previous practice within the NICU setting was informal assessment of PMH by MCHSW during their initial consult. MCHSW also attempted to contact NICU families weekly to offer support and determine the need for additional resources, but attempts were not always successful and were not formally tracked. For this initiative, when PMH concerns were detected through these interactions and referral for further evaluation and/or support was recommended, MCHSW recorded date of referral, parent sex, race, ethnicity, primary language, insurance type, history of mental health and/or PMH disorder, infant gestational age (GA), infant chronologic age at time of referral, referral type, and parent response to referral. Referrals included mental health providers, primary care providers, support groups, and peer mentors. We compared screening using validated tools to this previous practice of screening based on informal assessments to determine whether standardized, longitudinal screening was more effective in detecting mental health concerns.

**Fig. 1:**
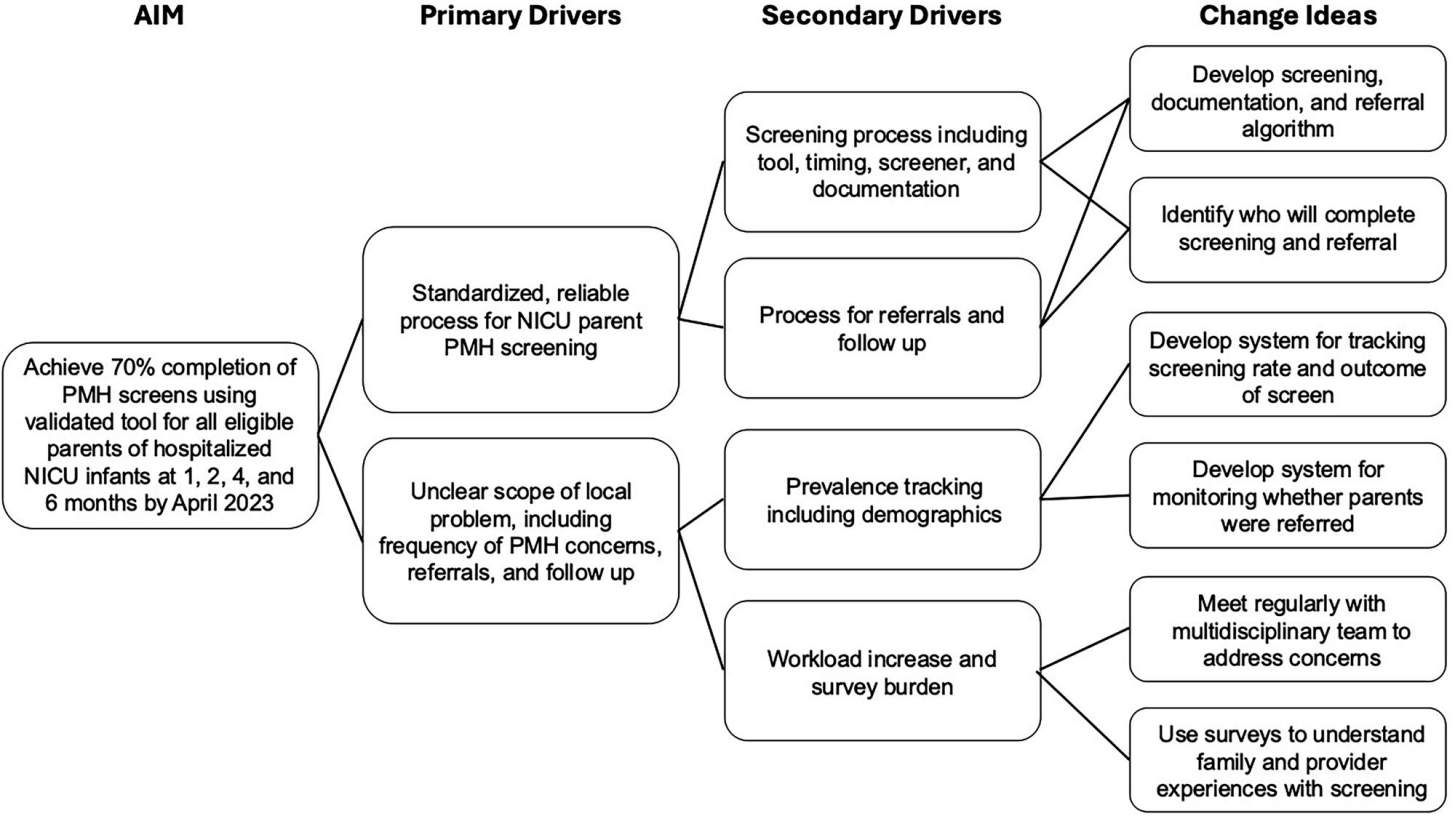
Driver diagram.

A multidisciplinary team inclusive of key stakeholders with diverse expertise, including former NICU parents, MCHSW, obstetrics, neonatology, and infant and child psychology, conceptualized change ideas through a driver diagram (Fig. 1) and developed screening algorithms (Supplemental Fig. 1). Hospital risk management approved our screening algorithm and documentation protocol. While we endeavored to create a system in which charts could be made for parents that did not have them, risk management approved the use of confidential notes within infant charts as these charts are not part of the record when responding to a request for medical records and are not accessible in patient portals, thus protecting parent privacy. The institutional review board determined this project to be not research involving human subjects as defined by DHHS and FDA regulations and not subject to institutional board review. The SQUIRE 2.0 guidelines provided a framework for this report.

### SMART AIM

We aimed to implement standardized, longitudinal PMH screening and achieve 70% completion of screening from a baseline of 0%, inclusive of mothers and partners, for eligible parents of hospitalized infants in the NICU at MHealth Fairview Masonic Children’s Hospital (MFMCH) at 1-, 2-, 4-, and 6-month intervals from October 2022 to April 2023.

### Setting and population

MFMCH is a 68-bed, level IV, academic NICU in Minneapolis, Minnesota. In 2023, 758 infants were admitted (Supplemental Table 1), the majority of whom were White (55%), non-Hispanic (87%), belonged to families with an English language preference (89%), and lived within the metropolitan area (87%).

In the screening period, parents were identified as eligible for screening weekly if they had an infant hospitalized at 1 month (31–37 days), 2 months (61–67 days), 4 months (121–127 days), or 6 months (181–187 days). Parents of infants who transferred to our unit were screened at the next eligible period or earlier if indicated per MCHSW. Partners were included if they were identified as a caregiver by the mother. Exclusion criteria included discharge within 6 days of meeting eligibility criteria or parents residing outside the United States, incarcerated, in inpatient mental health or substance use treatment, or in outpatient mental health treatment and screened previously by our team. Although parents who declined screening have been excluded in other initiatives [20], we included these parents in this study.

### Measures

The primary outcome measure was the rate of eligible parents undergoing PMH screening. Process measures were the percent of parents screening above thresholds concerning for clinical depression and anxiety, percent of parents who were appropriately referred based on scores above threshold, and percent of parents who declined screening or MCHSW involvement. These were chosen to ensure that high screening rates were not obscuring other issues with screening quality, including parent discomfort with disclosing PMH symptoms and inadequate referrals when concerns were detected. Balancing measures (Supplemental Table 2) included parent, MCHSW, and neonatologist perceptions of screening as burdensome to assess for unintended consequences of screening, including parent dissatisfaction or distress, lack of buy in from staff, or intolerable workload changes and were collected using a 2-question survey scored by 6-point Likert Scale with options to strongly agree/disagree, agree/disagree, or slightly agree/disagree.

To track measures, deidentified data, including date of screen, screening interval, EPDS and EPDS-3A scores, presence of suicidal thoughts, parent sex, GS, race, ethnicity, primary language, insurance type, history of mental health disorder and/or PMH disorder, infant GA, presence of older children in the family, referral type, and response to referral were recorded. For screens that were not completed, the date, screening interval, parent sex, GS, race, ethnicity, primary language, insurance type, infant GA, presence of older children in the family, history of mental health disorder and/or PMH disorder, and the reason that the screen was not completed were recorded. To facilitate anonymity of feedback, no demographics were associated with survey responses. Race, ethnicity, primary language, insurance type, GA, presence of older children, and mental health history were not analyzed.

### Interventions

After implementation of standardized, longitudinal PMH screening, MCHSW screened parents who met inclusion criteria for depression and anxiety using printed EPDS and EPDS-3A surveys. Screening was intended to be performed in person or over the phone to facilitate timely interventions for scores above threshold, but some families asked for screens to be left at bedside to be completed at their convenience. Two algorithms (Supplemental Fig. 1) were used, one for mothers (EPDS threshold of 10 [2], EPDS-3A threshold of 4) [26] and one for partners (EPDS threshold of 8 [27, 28], EPDS-3A threshold of 4 [29]).

Our response to scores above threshold for referral, psychosis, and suicidal ideation mirrored that of our institution’s outpatient policy on maternal depression screening at infant well child checks, which were similar in terms of timing of screens, thresholds for referral for mothers, follow up plans recommended, documentation that occurred outside of confidential notes in infant charts, and provision of patient educational material. Key differences in screening workflows are summarized in Table 1. In addition, acknowledging that partners may not have a primary care provider, we included contact information in our written protocol for internal medicine providers willing to evaluate and treat PMH concerns.

**Table 1:** Key differences in workflows between outpatient and NICU perinatal mental health screening programs.

	Well-Child Visit Screening Workflow	NICU Screening Workflow
Parents screened	Birthing mother	All parents
Symptoms screened	Depression	Depression, anxiety
Charts created for parents	Yes	No
Documentation	Parent chart	Parent chart if available; confidential note in infant chart if unavailable
Staff responsible for introducing screens	Front desk	MCHSW
Scripted introduction	Yes	No
Staff responsible for scoring screens	Medical assistant	MCHSW
Staff responsible for responding to screen and determining follow up plan	Pediatrician	MCHSW
Staff responsible for documentation of screening result and follow up plan	Pediatrician	MCHSW
CPT code used	Yes	No
Screens scanned into parent medical record	Yes	No

MCHSW documented screening results using a template in parent charts when available and in a confidential note in the infant chart when unavailable. If a referral was recommended but declined, we offered supportive check-ins and screened again at the next routine interval if their infant remained hospitalized.

Parent-facing educational materials and resources related to PMH were available on posters, informational cards, and surveys. Educational materials and surveys were translated into common language groups in our area including Spanish, Somali, and Hmong, and were reviewed by the Director for Diversity, Equity, and Inclusion and our QI team. Nursing and social work leadership and the neonatology division provided feedback prior to screening implementation.

Interventions are listed in Table 2. Practice changes that were incorporated into this initiative included greater attention to the PMH of partners, including assessment when partners were present at the initial MCHSW consult, discussion of PMH screening during the initial MCHSW consult, unit-wide education about PMH, changes to admission, progress, and discharge templates to include prevalence of PMH concerns during and after a NICU admission, and discussion of PMH needs during multidisciplinary health team rounds. These changes were not assessed systematically and are therefore not included in Table 2.

**Table 2:** Timeline of perinatal mental health interventions in the NICU.

Date	Intervention	Label^a^
October 2022	Screening initiated	A
November 2022	Educational cards with QR codes distributed at each screening interval	B
	Social work team lead leave of absence	C
December 2022	Educational posters displayed in lounges, bathrooms, pumping rooms, pump part rooms	D
January 2023	Social work team member leave of absence	E
March 2023	Check in with social work team with review of data and reminder to hand out informational cards	F
	Eliminated need for neonatologist to update screening section of daily progress notes	G
	Educational posters displayed on screens throughout NICU	H

^a^Labels are used to indicate the timing of interventions in Fig. 2.

### Data analysis

Improvement for primary outcome measure over time was analyzed in biweekly intervals and plotted using p-type statistical process control charts annotated with PDSA cycle interventions using QI Macros (QI Macros for Excel, version 2021; KnowWare International, Inc, Denver, CO). Chart type was selected due to data being discrete with one defect and an inconsistent subgroup size. Special cause variation was determined using Western Electric rules, with adjustment of median, upper, and lower control limits accordingly [30]. Analysis of process and balancing measures were completed using descriptive statistics.

## Results

### Primary outcome measure

**Fig. 2:**
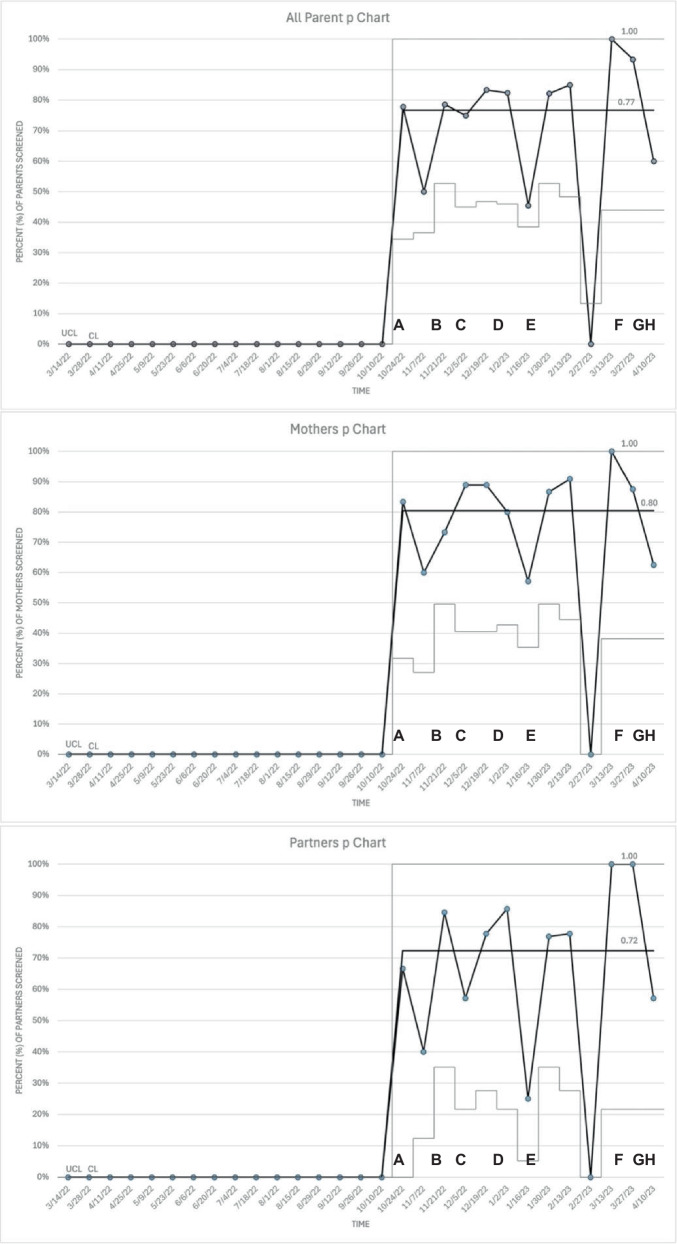
Statistical process control charts for all eligible parents (top), mothers (middle), and partners (bottom).

Our NICU standardized screening rates at baseline were 0%. In the first 6 months of screening, the average daily census was 60. 158/206 (77%; mothers 90/112 (80%), partners 68/94 (72%)) eligible parents were screened. 2/158 (1%; mother 1/90 (1%), partner 1/68 (1%)) screens were partially completed. The questions that were answered were scored to determine need for referral. Process change criteria was met after initiation of screening. The centerline for screen completion rate per week was 77% for all parents, 80% for mothers, and 72% for partners (Fig. 2).

Screen completion rates by screening interval for all parents were 85/116 (73%; mothers 48/63 (76%), partners 37/53 (70%)) at 1 month, 50/66 (76%; mothers 29/36 (81%), partners 21/30 (70%)) at 2 months, 17/17 (100%; mothers 9/9, partners 8/8) at 4 months, and 6/7 (86%; mothers 4/4 (100%), partners 2/3 (67%)) at 6 months.

There was an astronomical point noted on p-chart analysis when all parents were analyzed together. This occurred during a period when the MCHSW team was understaffed, and fewer parents were eligible for screening (4) compared to other periods (range 9-28).

### Process measures

During the 6-month pre-intervention period, 52 PMH referrals were made by MCHSW after an infant’s admission to the NICU. These referrals were primarily for mothers (49/52 (94%)). 38/52 (73%) referrals were made within 1 week after birth, and 12/52 (23%) referrals were made at or after 1 month after birth. 11/52 (21%) referred parents had established mental health providers. No concerns for suicidal ideation were noted based on informal assessments.

60/158 screened parents (38%) had scores above threshold for referral, including 42 mothers and 18 partners. By screening interval, 33/85 (39%; mothers 20/48 (42%), partners 13/37 (35%)) parents had scores above threshold for referral at 1 month, 15/50 (30%; mothers 12/29 (41%), partners 3/21 (14%)) parents at 2 months, 8/17 (47%; mothers 7/9 (78%), partners 1/7 (12.5%)) parents at 4 months, and 4/6 (67%; mothers 3/4 (75%), partners 1/2 (50%)) at 6 months.

6/158 parents (4%; mothers 4/90 (4%), partners 2/68 (3%)) disclosed passive suicidal ideation. Suicidal ideation was expressed in similar numbers across screening intervals. No parents disclosed active suicidal ideation.

46/60 (77%) parents with scores above threshold were referred according to the screening algorithm. Most missed referrals occurred at 1 month (10/14 (71%)), followed by 2 months (3/14 (21%)) and 4 months (1/14 (7%)). No referrals were missed at 6 months. Missed referrals were most frequent when EPDS and EPDS-3A scores were near the threshold for referral, including 8/14 (57%) instances for an EPDS-3A score of 4–5 and 6/14 (43%) instances for partners with an EPDS score of 8–9. 1/98 (1%) parents with a screen below threshold for referral was referred based on clinical concerns after screen completion. Of the parents referred, 12/47 (26%) parents had established mental health providers.

4/206 (2%) parents were not screened because they declined screening (*n* = 1), declined social work involvement (*n* = 2), or returned blank screens (*n* = 1). This represented parents within 3 families. The parent who declined screening cited privacy concerns and lack of time to complete screen as a reason for declining.

### Balancing measures

**Fig. 3:**
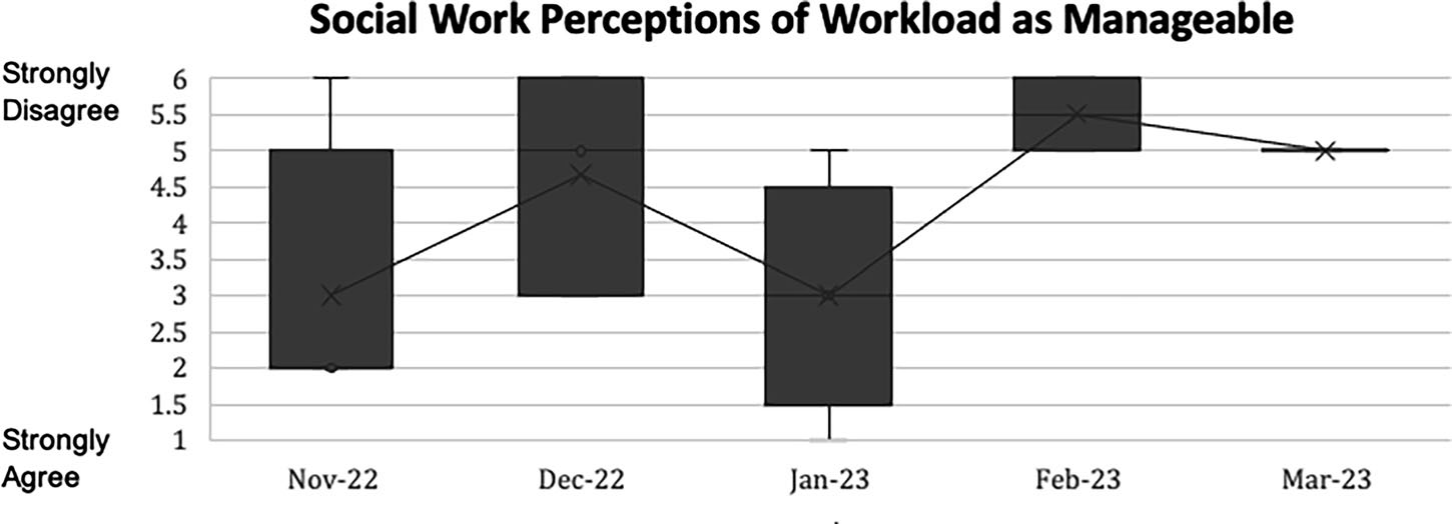
Distribution of answers to survey questions of social work perceptions of workload as manageable over time.

140/206 (68%) parents returned completed or partially completed surveys. Screening was not identified as valuable by 10/140 (7%) parents. Screening was identified as difficult by 15/139 (11%) parents. No neonatologists or MCHSW identified screening as not valuable. MCHSW unanimously and 51/57 (89%) of neonatologists chose “Strongly agree” when asked to agree or disagree that standardized PMH screening was valuable. The workload associated with screening was identified as unmanageable by 2/57 (4%) neonatologists and 8/15 (53%) MCHSW. For MCHSW, workload was perceived as less manageable over time (Fig. 3).

## Discussion

PMH screening rates in published QI initiatives within NICUs have increased from a baseline of 0–28% to 22–99% through targeted interventions, though inclusion criteria for each screening program varied [22]. The primary aim of this study was to increase our PMH screen completion rate for parents with infants hospitalized in the NICU at 1, 2, 4, and 6 months from a baseline of 0% to 70% within 6 months. We were able to achieve our aim for mothers and partners. This QI initiative is unique as it compares standardized screening to our previous practice of informal assessments of PMH alone, involves parents on the QI team, assesses parent and provider perceptions of screening, and includes comparable numbers of partners. It is in the minority of published QI initiatives on PMH screening that include symptoms beyond depression and perform screening longitudinally [22]. Based on our study observations, we have outlined five key considerations for hospital-based QI teams in developing PMH support programs for parents impacted by the NICU.

First, a pre- and post-intervention cohort comparison demonstrated that standardized PMH screening compared to informal assessments alone improves detection of PMH concerns in NICU parents beyond the first few weeks of hospitalization, when interactions with the social work team may be less frequent. Although our numbers of parents with NICU stays 4 months or longer were low, we found that standardized, longitudinal screening programs may be especially important considering the increased proportion of mothers with screens above threshold for referral at 4 months and parents with screens above threshold for referral at 6 months. Our comparison of standardized screening to informal assessments is important because the majority of NICUs report using informal assessments alone or in combination with formal screening [31]. Our results support recommendations to perform universal screening that is standardized using validated tools [5].

Questions regarding when to initiate screening and when to repeat screening remain. Many published initiatives screen at 14 days and do not repeat screening [22], despite evidence that PMH symptoms may peak later than this [2]. Our experience that informal assessments most frequently detect PMH concerns within the first few weeks of a NICU stay, as well as existing evidence that some of the concerns identified in the first two weeks of an infant’s birth do not persist [3, 6], support decisions to initiate screening in the NICU at the first AAP-recommended interval for outpatient screening, rather than an earlier time. Furthermore, recommendations for early screening soon after admission [5, 32], which recognize that many infants have NICU stays shorter than 14 days, predate the most recent AAP policy statement on postpartum depression screening at infant well child visits [2]. Thus, PMH screening for mothers impacted by shorter NICU stays is likely to occur within well child visits, although quality of local outpatient screening may vary, it is unlikely to include partners, and screening is often limited to depression symptoms. Partnership with primary care practices, which often have fewer embedded psychosocial supports, as well as NICU neurodevelopmental follow up clinics is essential to ensuring quality PMH screening occurs for families after NICU discharge. Future research is needed to understand the optimal intervals and duration of screening for NICU parents.

The second consideration is that standardized screening of all parents is important. Previous initiatives were not able to screen mothers and fathers with similar frequency [24, 33]. We found that standardized, longitudinal PMH screening increased the percent of PMH identified in partners fivefold, suggesting that the needs of partners are not met without systematic attention to their inclusion within the NICU. Efforts should be made to ensure screening and educational materials allow all parents to feel included. Specific local peer support groups for NICU fathers and LGBTQ+ parents may be beneficial.

Inclusion of partners in screening programs may compound concerns about documentation of screens, as partners may be less likely to have patient charts within an institution’s electronic medical record. In discussions with risk management and legal experts regarding federal and state-specific privacy laws, it was determined that documentation of screening results in confidential notes within the infant chart safeguards parent privacy by preventing parents’ screening results from being disclosed to unintended recipients through patient portals or requests for medical records. Still, best practice is to create separate parent charts for documentation of screening results. Discussions with risk management and privacy teams as well as partnership with outpatient providers to align screening practices locally is an important component of screening implementation at the institutional level.

The third consideration is that a dedicated workforce for PMH screening is needed for successful screening implementation and follow up. Designated staff for screening, referring, and auditing PMH screening programs has been identified as a key component in other studies [22], but barriers in creating such positions at the local level remain. Moreover, while leaders in PMH have recommended psychotherapy within the NICU for parents at highest risk [34] and that all NICUs with 20 or more beds should have at least one full-time or part-time doctoral level psychologist [5], this recommended infrastructure remains aspirational within many systems [31].

The expertise of MCHSW is therefore needed to support the PMH needs of parents in many NICU settings. Despite valuing screening, the workload burden endorsed by MCHSW in our unit progressed over time. When the burden of screening was discussed at routine check ins, MCHSW declined trialing alternative screeners, as they believed screening would be manageable once they were fully staffed. Still, the increased workload associated with PMH screening should not be underestimated, as quality screening takes time to perform, and parents do not value screening that feels ineffective [13]. We used printed screens to limit privacy concerns. However, electronic screens may improve workload and should be considered in collaboration with parent experts.

The fourth consideration is that in contrast to what has been described in initiatives which only screen for maternal postpartum depression [20], our screening algorithm was not followed perfectly. Errors in determining appropriate follow up plans were likely related to our use of two screens (EPDS and EPDS-3A) as well as our use of different thresholds for mothers and partners. Electronic screening with automated scoring may improve algorithm adherence when multiple tools or thresholds are used. Further work is needed to determine optimal thresholds for referral for NICU families.

Finally, we posit that involving a multidisciplinary team inclusive of parent experts to inform local screening guidelines may improve program success. We noted fewer parents declined screening in our program compared to other published programs [35]. It is likely that the quality of screening, including discussion of how results will be used to support parents [24], impacts parent perceptions. As an alternative to separate surveys to gain parent perspectives on screening, tracking the percentage of screens declined and the reasons why may prove beneficial. Further work across institutions should establish a benchmark for the expected rate of declined screening and endeavor to understand which parents decline screening and why.

Limitations of our baseline data include that data collection was performed by MCHSW and may underestimate the number of referrals made based on informal assessments or may overestimate PMH concerns detected through informal assessments due to observer bias. We did not measure how many parents had PMH concerns detected through standardized, longitudinal screening that would have been missed through informal assessments alone. Moreover, given the increased workload associated with screening, we were not able to track referrals made based on informal assessments after implementing screening. However, based on discussions with MCHSW, we presume that the number of referrals based on informal assessments was similar in both periods. We were unable to track follow up of mental health concerns in the initial PDSA cycles but found 35/47 parents (74%) verbally agreed to a referral made after screen completion, indicating at least an intent to follow up. We suggest that future PMH screening implementation initiatives consider a mechanism to more accurately track the number of parents with PMH concerns detected through clinical interactions versus screening, the percent of parents in the NICU who have PMH concerns, the percent of parents whose concerns were only detected through screening, and the percent of parents who receive follow up mental healthcare after referral based on informal or formal assessments to better understand and improve PMH screening programs.

Other limitations of this work include persistent variability in screening rates. Based on the increased variability of screening observed during periods associated with decreased MCHSW workforce, we posit that this was related to staffing issues, a barrier that was not always able to be overcome despite MCHSW dedication to screening. There was an astronomical point at which no eligible parents (1 mother, 3 partners) were screened. The ultimate reason for this point is unclear as screening rates remained above goal during other weeks while understaffed. Although comparatively fewer parents were eligible for screening and therefore theoretically the workload associated with screening was less, partners are often not able to spend as much time in the NICU and may be more difficult to reach by phone due to work schedules. Lastly, while we recognize concerns regarding the equity of PMH screening programs within NICUs [16, 20], in this analysis, we did not assess for differential impacts of screening. These should be assessed and targeted in future work.

We continue to develop change ideas, including the use of electronic screening and increased unit-wide engagement with PMH, that will result in reliable, sustained screening that is effective in connecting parents to resources and that is less dependent on staffing, while attempting to preserve desired ownership of MCHSW over screening. Increased variability in screening associated with decreased MCHSW workforce and fewer than half of MCHSW identifying the workload as manageable supports the need for expansion of MCHSW workforce within our NICU to account for the extra workload associated with screening. In addition, embedded psychology support to assist families through their NICU admissions and beyond likely benefits families, as other QI initiatives have observed high follow up rates when counselors are embedded within the NICU at no cost to families [19]. This work has facilitated the development of interventions involving infant mental health teams for dyadic support of hospitalized infants and their families within our NICU. Future work is needed to understand and decrease barriers to PMH treatment, including the cost of mental health visits, and promote best practices across systems for parents in NICUs without embedded psychology support. Further research should assess the benefits and burdens of screening only for depressive symptoms versus including other PMH symptoms as well as optimal screening tools for parents impacted by the NICU, especially for partners and inclusive of LGBTQ+ parents. We suggest that national workgroups partner with parent experts to establish measures, benchmarks, and standardized exclusion criteria to assist individual PMH screening programs in defining program success.

## Supplementary information

Supplemental material

## Data Availability

The dataset generated during this initiative is available from the authors upon reasonable request.
