# Supplementary material for: Implementation of perinatal mental health screening for parents of infants in a level IV neonatal intensive care unit: A quality improvement initiative
Source: J Perinatol. 2025 May 7;45(6):859–66. doi: 10.1038/s41372-025-02315-z (PMC12263419; doi:10.1038/s41372-025-02315-z)
Supplement: Supplementary file 1 — Supplemental material [file 41372_2025_2315_MOESM1_ESM.docx]

Supplemental materials

**Supplemental Table 1** Characteristics of admitted infants to Level IV NICU in 2023

| Demographic | Infants (%) |
| --- | --- |
| Race | 758 |
| American Indian or Alaska Native | 18 (2) |
| Asian | 59 (8) |
| Black or African American | 140 (18) |
| Multiracial | 37 (5) |
| Native Hawaiian or Other Pacific Islander | 1 (0) |
| White | 415 (55) |
| Unknown/Other | 88 (12) |
| Ethnicity | 758 |
| Hispanic or Latino | 68 (9) |
| Not Hispanic or Latino | 661 (87) |
| Unknown/Other | 29 (4) |
| Birth Weight | 758 |
| Extremely Low Birth Weight | 79 (10) |
| Very Low Birth Weight | 58 (8) |
| Low Birth Weight | 201 (27) |
| Normal Birth Weight | 355 (47) |
| Unknown | 65 (9) |
| Preferred Language | 758 |
| Somali | 29 (4) |
| Spanish | 30 (4) |
| English | 676 (89) |
| Distance from home | 758 |
| Local | 662 (87) |
| Within state | 75 (10) |
| Out-of-state | 21 (3) |

**Supplemental Table 2** Items on parent, social work, and neonatologist balancing measure surveys

|  | Items on Survey |
| --- | --- |
| Parent | Mental health screening for NICU parents positively impacts the health of myself and my baby. |
|  | I found it difficult to complete this questionnaire. |
| Social Work | Routine screening of parents in the NICU for perinatal mood and anxiety disorders is valuable. |
|  | The workload associated with routine mental health screening for parents in the NICU is manageable. |
| Neonatologist | Routine screening of parents in the NICU for perinatal mood and anxiety disorders is valuable. |
|  | The workload associated with routine mental health screening for parents in the NICU is manageable. |


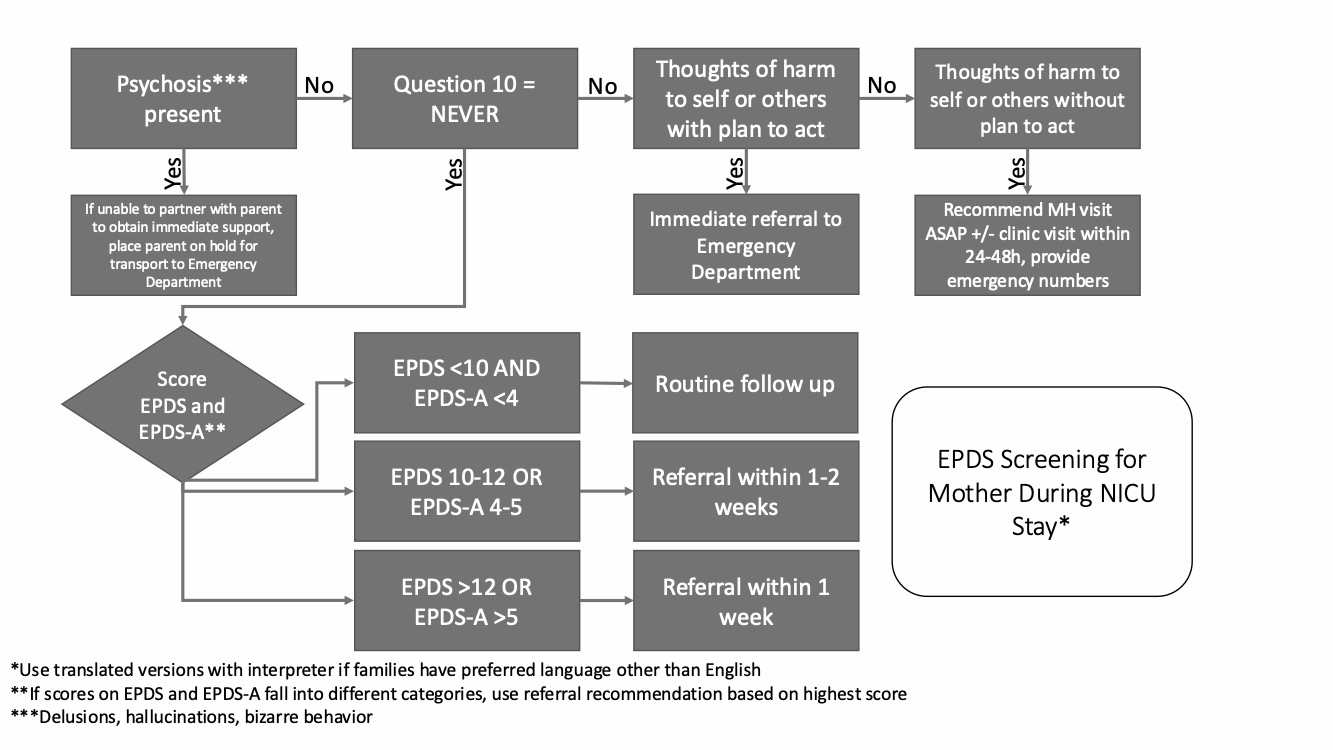


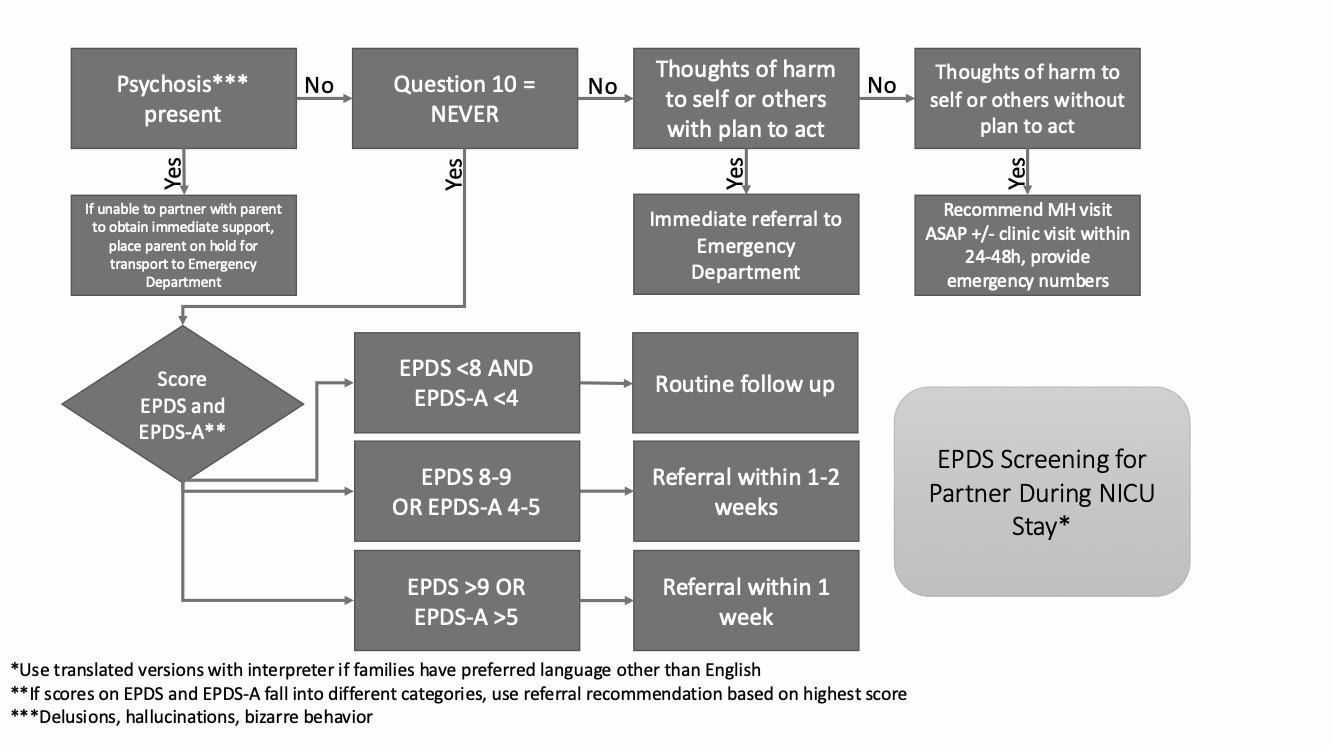


**Supplemental Figure 1** Screening algorithm for mothers (top) and partners (bottom)
